# Utilisation of Healthcare and Associated Services in Huntington’s disease: a data mining study

**DOI:** 10.1371/currents.RRN1206

**Published:** 2011-01-21

**Authors:** Monica Busse, Dr. Hasan Al-Madfai, Joyce Kenkre, G. Bernhard Landwehrmeyer, AnnaRita Bentivoglio, Anne Rosser, The European Huntington’s Disease Network

**Affiliations:** ^*^Department of Physiotherapy, Cardiff University, Ty Dewi Sant, Heath Park, Cardiff, UK; ^†^BGL Group; ^‡^Health, Sport and Science, University of Glamorgan Pontypridd UK; ^§^Universitätsklinikum Ulm, Neurologie; ^¶^Istitutodi Neurologia, Istituto di neurologia, Università Cattolica del Sacro Cuore, Roma, Italy and ^#^Cardiff University

## Abstract

Background: People with Huntington’s disease (HD) often require tailored healthcare and support packages that develop as the disease progresses. The Client Service Receipt Inventory (CSRI) gathers retrospective information on service utilization. This study investigated the use of formal services and informal care as measured by the CSRI and explored associations between informal care, disease severity and functional ability as measured by the Unified Huntington’s Disease Rating Scale Total Motor Score (UHDRS-TMS) and functional scales.

Methods: All monitored longitudinal data from annual clinical assessments of UHDRS-TMS and functional assessments and CSRI collected under the auspices of the European Huntington’s Disease Network (EHDN) REGISTRY study between the years 2004 and 2009 were utilised in the analyses. Disease severity was reflected by UHDRS-TMS. Functional ability was measured using the UHDRS functional scales. CSRI data were analysed according to percentage use of individual formal services and total estimated hours per week of informal care. Regression analyses were conducted to identify any associations between disease severity, functional ability and hours of informal care.

Results: 451 HD patients (212 female; 239 male) completed one visit; 105 patients (54 females; 51 males) completed two visits and 47 patients (20 females; 27 males) completed three visits in total over the 5 year period. The mean time between visits was 1.2 years. At visit one, 74% of the participants reported being in receipt of at least one formal hospital-based service in the previous six months, and 89% reported receipt of formal primary and community care services. In contrast, at the third visit, 62% of people had used hospital based services and 94% formal community based services in the previous six months. Fifty % of individuals required some form of informal care in the home at visit 1; this increased to 68% at visits 2 and 3. The mean (SD) estimated weekly total informal care hours at visits 1, 2 and 3 were 32.8 (49.4); 21.6 (53.6) and 21.3 (62.4) respectively. Only the scores on the Functional Assessment Scale (FAS) accounted for the variance in the weekly total informal care hours at each visit.

Conclusions: Although it must be acknowledged that service use is supply driven, most HD patients across Europe surveyed as part of this study were in receipt of formal primary and community care services and to a lesser extent formal hospital based services. There was however a large reliance on informal care in the home. The FAS appear to have predictive value on informal care requirements and may have utility in facilitating pro-active service provision and in particular when managing carer burden in this population.

## **Introduction:**

Huntington’s disease (HD) is an inherited, neurodegenerative disease resulting in clinical symptoms of progressive movement disorder, cognitive defects and behavioural changes that ultimately affects a person’s ability to participate independently in activities of daily living, work and community. With an approximately 20 year time course from symptom onset, death usually results from complications of falls, dysphagia, or aspiration [1]. Over the course of the disease, the person with HD will require a range of generic and specialist professional services [2] [3]and flexible, co-ordinated practical and psychological support [4] depending on the stage of condition and that person’s functional status. In the earlier stages of the disease, healthcare focuses on maintaining and/or preventing decline in mental and physical functioning. Later on in the disease, care is usually more supportive in nature. Although cognition and behavioural problems have a greater incidence in people requiring full time care, advanced motor impairment has been found to be the greatest predictor of nursing home placement in HD patients [5].  

Successful care will usually involve the efforts of a multi-disciplinary team working closely with the family to optimize quality of life over the life span of the disease [6] [7] [8]. Here, care of the person with HD should be person-centred with the person with HD and their immediate family linked to the multi-disciplinary team for appropriate medical, health and social care so as to maximize function and prepare for future challenges [3]. Health professionals involved in the care of the person with HD may include the General practitioner, Psychiatrist, Neurologist, Geneticist, Psychologist, Social Worker, Speech Therapist, Occupational Therapist, Physiotherapist and Community and/or specialist nurse [6]. Social services and home help or nursing may also be crucial. The diverse nature of symptoms seen in different individuals with HD, over time and involving various services make care planning and service development complex [6]. There have been suggestions that due to these complexities, in many cases, care needs are unmet [6] [9]both in the earlier [10] and more palliative stages of the disease [8]. The complexity in provision of care requirements also often results in the family members i.e. informal carers undertaking the overall responsibility for care [11]. 

Informal care is that which is delivered by non-expert carers who are usually family or friends, may range from full time care from person who lives with the HD patient to intermittent input from someone living elsewhere.  Informal care will vary according to the needs of the person with HD and may range from looking after all personal needs to help with shopping and care of home on less frequent basis. Informal carers are often required to provide both psychological and practical support over extended periods of time [4] and the impact on their quality of life as a result of supporting the person with HD at home should certainly not be underestimated [12] [13]. Informal care is also not subject to audit or regulation and may not always be optimal for the person with HD and for the person providing that care. 

To be able to ensure that care needs are met, appropriate and informed service planning is required. The first step in this process is to quantify actual use of formal healthcare resources and the balance between formal care services and informal care use. 

Knowledge of such data has the potential to facilitate appropriate planning and resource allocation in line with standards of excellence for chronic conditions management. In the United Kingdom, a National Service Framework for people with Long Term Neurological Conditions (LTNCs) sets out quality requirements for care. These include prioritisation for personal care and support with appropriate support for family and carers and access to appropriate aids and home adaptations to promote living at home to facilitate living at home for as long as is able with as high a quality of life as possible [14]. In order to evaluate whether such quality standards are being met, current service use needs to be described. There is an on-going programme of study evaluating service use in rare LTNCs (RESULT study) (http://www.ltnc.org.uk/research_files/RESULT_study.html) for this specific purpose but to date no data is available in published literature of specific service receipt in HD patients.  

The Client Service Receipt Inventory (CSRI) questionnaire was developed [15] [16] specifically to provide information on service utilisation and ultimately inform service delivery by collecting retrospective information on service related issues. It is an established tool and has been used in a range of research studies including mental health outreach services, community nursing services and community care of older people and people with challenging behaviour [16] [17]. The questionnaire schedule is designed for interviewer/researcher administration with the person receiving the services assisted by their main carer as required. The retrospective period is fixed according to the population being investigated and according to the specific aims of the research. It therefore varies by the research design in question and allows capture of information on rarer services that may be received in a set time period whilst not being subject to the problems of recall bias. The service receipt section is the core of the CSRI [16]; for each service type, the number and average duration of contacts is recorded and used to summarise particular care packages, illustrate the variety of services used and in particular determine how resources are allocated.  HD patients at different stages of the disease will have particular health care needs requiring specific combinations of health services and this will have associated service planning implications. Clear assessment of disease impact and severity alongside healthcare resource use is a key requirement for resource planning and predicting future care needs. 

Disease severity is routinely quantified using the Unified Huntington’s Disease Rating Scales (UHDRS) [18] [19], a disease specific rating system that is used to assess the severity of disease. With the UHDRS, specific domains of clinical performance namely motor ability, cognition and behaviour are assessed. The motor rating involves 31 questions representing oculomotor, speech, motor, and gait findings rated on a 0- to 4-point scale (4 most impaired). The cognitive assessments incorporate verbal fluency, ability to pair numbers with figures and selective attention whilst the behavioural assessment considers severity and frequency of mood, anxiety, aggression, psychosis, and other behavioural abnormalities. The UHDRS functional components comprise three ordinal components, namely the functional assessment scale (FAS) (an activities of daily living checklist with range 0 to 25), the Independence scale (IS) (range 10-100), and the Total Functional Capacity (TFC) (range 0 to 13). Scores on the TFC can further represent five stages in the neurodegenerative disease process: stage I represents scores from 13-11; stage II, scores of 10-7; stage III, scores of 6-3; stage IV, scores of 2-1; and stage V, a score of 0. The independence scale has a range of 10 to 100 units and evaluates dependence on care from total care required to no special care needed.

The aim of this study was to investigate the use of formal and informal care services as recorded using retrospective CSRI data and to investigate any associations between use of care, disease severity and functional ability as measured by the UHDRS motor and functional scales. 

In these analyses, we specifically sought to identify, a) what care (both formal services and informal care) HD patients currently use and b) whether there were any specific factors associated with the estimated amount of informal care required. To our knowledge such data has not previously been presented for a cohort of HD patients; we suggest that availability of such data in a cohort of HD patients from a cross-section of European countries is of use in quantifying and ultimately improving planning of service provision for HD patients.   

**Methods and materials:**

REGISTRY is a multi-centre, multi-national observational study with no experimental intervention. REGISTRY is managed by the European Huntington’s Disease Network (EHDN) and aims to build up an electronic database of patients with HD and their relatives. The main component of the ' REGISTRY ' study is the storage of anonymous medical data on an electronic database. One of the aims of REGISTRY is to obtain natural history data on an annual basis over an extended time period on a wide spectrum of individuals affected by HD to facilitate data mining studies. 

For the purposes of the analyses presented here, the monitored data of demography, CAG repeat, estimated age of onset, disease severity and functional ability as measured by the Unified Huntington’s Disease Rating Scale total motor score (UHDRS-TMS) and functional scales in combination with the CSRI has been utilised for data mining. Data was collected according to the standard REGISTRY protocol [20].  The EHDN REGISTRY CSRI refers to the immediate six month retrospective period. It records details on use of hospital and residential services; primary and community care services; investigations and diagnostic tests; aids or devices; adaptations to the home; informal care; average loss of income per week; journeys undertaken and occupation. 

Data were analysed descriptively for demographics and according to percentage use of individual services and aggregates of service receipt namely 1) total number of individual formal services received 2) aggregates of hospital services (inpatient and outpatient) and primary and community care and 3) the carer or patient estimated total informal care hours (personal, help inside the home, help outside the home and other) as recorded by the CSRI. As the focus on our investigations was to establish service use frequencies in the first instance and not costs per se, we do not report on investigations and diagnostic tests, average loss of income per week; journeys undertaken and occupation in these analyses.

In order to identify specific factors associated with receipt of services at each visit, we utilised linear regression with estimated hours of informal care as the dependant variable and the measures of disease severity and functional ability as the independent variables.  All analyses were conducted using PASW version 18 Release 18.0.2 April 2010. 

## **Results**

### **Demographic and Descriptive data:**

CSRI data for 451 HD patients (212 female; 239 male) was available for visit one (at enrolment to the study) of this observational study. Completed CSRI data for 105 (54 females; 51 males) and 47 (20 females; 27 males) HD patients was available for visits two and three respectively. The mean time between visits was 1.2 years. Countries represented were Austria (8.6%); Germany (33.7%); Italy (15.3%); Norway (3.3%); Poland (18.4%); Portugal (2.9%); Spain (10.6%); Switzerland (0.9%) and United Kingdom (6.2%). 

Mean (SD) estimated age of onset was 41.8 (12.8) years and age at visit 1 was 49.5 (13.1). Median (range) CAG repeat length was 45 (35-89). Median (range) scores at visit 1 on UHDRS-TMS, Functional assessment scale (FAS), Total Functional Capacity (TFC) and Independence Scale were 34 (0-106); 20 (0-25); 8 (0-13) and 80 (10-100) respectively. At Visit 2, median (range) scores on UHDRS-TMS, Functional assessment scale (FAS), Total Functional Capacity (TFC) and Independence Scale were 37 (0-111); 16 (0-25); 7 (0-13) and 75 (10-100) respectively.At Visit 3, median (range) scores on UHDRS-TMS, Functional assessment scale (FAS), Total Functional Capacity (TFC) and Independence Scale were 43 (0-97); 13 (0-25); 5 (0-13) and 70 (25-100) respectively. It is important to note the altered proportional representation of people with HD at each stage of the disease for each visit. At visit 1, most participants (n=153; 34%) were at Stage 1 of the disease; by visit 3 most participants (n=58; 61%) were at either stage 3 or 4 of the disease. Table 1 gives details of percentage of the sample at each stage of the disease for each visit.  

**Table 1: Frequencies and valid percentages of HD patients at each visit stratified according to stage of disease (represented by TFC scores)**

**Table:** 

TFC stage of disease	Number (%) of total sample at visit 1 at each stage of the disease	Number (%) of total sample at visit 2 at each stage of the disease	Number (%) of total sample at visit 3 at each stage of the disease
1 (TFC 11-13)	153 (34%)	59 (27%)	18 (19%)
2 (TFC 7-10)	125 (28%)	57 (25%)	18 (19%)
3 (TFC (3-6)	119 (26%)	74 (33%)	36 (38%)
4 (TFC 1-2)	47 (10%)	31 (14%)	22 (23%)
5 (TFC 0)	7 (2%)	3 (1%)	1 (1%)

### **Receipt of services:**

At visit 1, 335 of the 451 participants were in receipt of at least one formal hospital based service and 401 (89%) were in receipt of primary and community care services. In contrast, 94% of people used community based services at visit 3 and 62% used hospital based services. Fifty percent of individuals reported use of informal care in the home at visit 1; this increased to 68% at visits 2 and 3 (see Table 2).The increase in service use frequencies and care hours reported in visits 2 and 3 do however need to be considered in light of the greater proportion of people in stages 3 and 4 of the disease at visit 3 compared to those at visit 1 (see Table 1).  

Inpatient hospital service use increased dramatically over visits and outpatient service use decreased. Percentage of people in receipt of physiotherapy and speech and language therapy services increased at each visit; whilst social worker involvement remained relatively constant. Home help use increased over time. Notable is that occupational therapy, dentistry and other allied health professional services was not independently recorded in the version of CSRI used here and may well be under-reported in this situation. There was additionally no mention of key-worker involvement.      

**Table 2: Frequencies and valid percentages of HD patients receiving care services at each visit**

**Table:** 

Service receipt for HD patients	Number (%) of total sample receiving services at visit 1 (n=451)	Number (%) of total sample receiving services at visit 2 (n=105)	Number (valid %) of total sample receiving services at visit 3 (n=47)
**Hospital services ** Inpatient Outpatient	335 (75%) 110 (24%) 300 (69%)	73 (70%) 58 (64%) 17 (16%)	29 (62%) 28 (60%) 5 (11%)
**Primary and Community care** GP Neurologist Other doctor Physiotherapist Social Worker Nurse Speech Therapist Home-help Other (Occupational therapy, Dentistry; Chiropractor)	401 (89%) 333 (74%) 170 (38%) 73 (16%) 91 (20%) 45 (10%) 30 (7%) 58 (13%) 51 (11%) 42 (9%)	89 (85%) 68 (65%) 35 (33%) 14 (14%) 31 (30%) 14 (13%) 15 (14%) 18 (17%) 15 (14%) 15 (14%)	44 (94%) 36 (77%) 25 (53%) 6 (13%) 21 (45%) 4 (9%) 4 (9%) 13 (28%) 9 (19%) 7 (15%)
**Informal Care ** Personal care Help inside the home Help outside the home Other	224 (50%) 130 (29%) 195 (43%) 170 (38%) 36 (8%)	71 (68%) 14 (39%) 63 (60%) 54 (51%) 14 (13%)	32 (68%) 22 (47%) 29 (62%) 25 (53%) 7 (15%)

### **Use of aids and adaptations:**

The use of wheelchairs increased over each visit, but all other appliance use remained fairly constant (see Table 3). Crutches and frames were used fairly infrequently whilst home adaptation (bathroom, shower and toilet) was a slightly more frequent occurrence.     

**Table 3: Frequencies and valid percentages of HD patients using aids and adaptations in the home at each visit**

**Table:** 

Aids and Adaptations in the home	Number (%) of total sample at visit 1 (n=451)	Number (%) of total sample at visit 2 (n=105)	Number (valid %) of total sample at visit 3 (n=47)
**Aids or devices** Wheelchairs Crutches Walking frames/ Rollators Other **Adaptations to the home** Stair lift Shower/bath relocation Toilet relocation Redesign kitchen Medicalised Bed Concrete ramp Other (Move home)	27 (6%) 10 (2%) 17 (4%) 17 (4%) 11 (2%) 26 (6%) 17 (4%) 7 (2%) 8 (2%) 3 (1%) 31 (7%)	17 (16%) 1 (1%) 7 (7%) 5 (4%) 4 (4%) 13 (12%) 7 (7%) 3 (3%) 5 (5%) 3 (3%) 14 (13%)	17 (15%) 2 (4%) 1 (2%) 5 (11%) 1 (4%) 11 (23%) 4 (9%) 1 (2%) 1 (2%) 0 (0%) 2 (4%)

### **Aggregates of Service Use:**

The mean (SD) estimated weekly total informal care hours at visits 1, 2 and 3 were 32.8 (49.4); 21.6 (53.6) and 21.3 (62.4) respectively (see Table 3). Although the mean reported informal care hours were less at each visit; the mean number of informal care services used appeared to increase between visits 1 and 3. Mean number of formal primary and community services used (and percentages of individual services) also appeared to increase over time whilst formal hospital based services reduced over time. This is consistent with what was seen in terms of percentages of HD patients using different services (see Table 2).   

**Table 4: Aggregates of service use for each visit**

**Table:** 

	Mean (SD); range of total individual formal service receipt	Mean (SD); range of number of hospital based services used	Mean (SD); range of number of primary and community care services used	Mean (SD); range of number of informal care services used	Mean (SD) informal care hours
Visit 1 (n=451)	3.1 (1.8); 0-9	1.1 (0.9); 0-5	2.0 (1.4); 0-7	1.2 (1.4); 0-4	32.7 (49.4)
Visit 2 (n=105)	3.2 (2.2); 0-10	1.1 (1.0); 0-4	2.1 (1.5); 0-7	1.6 (1.4); 0-4	21.6 (53.6)
Visit 3 (n=47)	3.5 (1.7); 0-10	0.8 (0.9); 0-4	2.6 (1.5); 0-8	1.8 (1.4); 0-4	21.3 (62.4)

### **Predictors of informal care requirements:**

Only the scores on the FAS accounted for the variance in the weekly total informal care hours at visit 1 (see Table 4). This remained a constant pattern over time. At Visit 2, the Functional Assessment Scale accounted for 9 % of the variance in hours of informal care (adjusted R^2^ 8.6%; SE 59.9; F7.8 p<0.007) (excluded variables: TFC, UHDRS-TMS and Independence Scale). At Visit 3, the Functional Assessment Scale accounted for 33 % of the variance in hours of informal care (adjusted R2 30%; SE 25.7; F 13.6; p<0.001) (excluded variables: TFC, UHDRS-TMS and Independence Scale).     

**Table 5: Linear regression on estimated hours of informal care at Visit 1**

**Table:** 

Independent variables	Standardized coefficients	t	Significance
Constant			
Functional Assessment Scale	-0.428	-3.423	0.000

Adjusted R^2^ 0.181 ; SE 34.186; F 88.784; p< 0.0001; dependant variable weekly total informal care hours    

## **Discussion**

Consideration of service use data reflecting current utilisation in the included sample is an important first step in the provision of high quality care. This study employed the CSRI to gather information about types and quantities of resources used by HD patients. We did not attempt to provide any indication of the monetary cost of service utilisation.  This is partly because unit costs will vary according to country and hence in some cases will be applied only to small numbers, but mainly because such “cost of illness” information would not necessarily be optimal in informing policy decisions. Quantifying costs of services (rather than demonstrating frequencies of resource use as we have done) would be more appropriate in the context of an economic evaluation of a specific intervention [21]. 

Most HD patients surveyed as part of this study were in receipt of some formal primary and community care services and to a lesser extent hospital based services. Trends in service provision over time may be inferred from the longitudinal data presented but it is important to acknowledge that some bias may be introduced to the study simply as result of being a research participant in a large scale observational study such as REGISTRY. In addition, as the numbers of participants decrease with each visit, there is an associated change in proportional representation of people at each stage of the disease. We cannot infer that the cohort is deteriorating over time resulting in an increased service use; the changes in service use may simply be skewed by this proportional change in individuals at each stage of the disease. We therefore suggest that a general impression of service receipt is best judged at visit 1 (study entry) where any effect on reporting rates and the bias introduced by the annual visit or the altering proportional representation of the sample at each visit is limited. 

At enrolment to the REGISTRY study, 89% of HD patients assessed in this data mining study were in receipt of at least 1 form of primary or community care service. It is not possible to establish any measure of how integrated these services were and additionally there was no clear indication of key worker or occupational therapy involvement as this was not part of the standard questions employed in the REGISTRY CSRI. Social worker and nurse involvement was fairly minimal and use of formal home help was reported in only 11% of participants in this cohort. Approximately 1/5th of participants were in receipt of physiotherapy services at the first visit and the use of crutches and walking aids was infrequent. Such infrequent use of walking aids is congruent with suggestions in the literature that physiotherapists do not recommend routine use of aids as choreic movement and cognitive impairment may influence safe use of walking aids in HD patients [22]. 

When considering service use frequencies, it is useful to compare with other groups of patients with neurodegenerative diseases. A report (published in 2000) of community services for people with Multiple Sclerosis (MS) (n=150) living in the United Kingdom found that at that time of 45% of people with MS did not receive any form of community services [23]. The remaining 55 % of people with MS were in receipt of a range of services including home help (20%), occupational therapy, physiotherapy (23%), social work (10%) and district nursing (17%). Twenty-seven % of participants had bath or shower adaptations in place. Those people who had no home adaptations (42%) in place were also not in receipt of any services. There appeared to be a substantial unmet need for people with MS in terms of community care with little change since the beginning of the previous decade[23]. The authors advocated the need for integrated, co-ordinated and multi-agency community service for people with MS. Of concern in our data (in light of the suggested relationship between lack of integrated community care and lack of home adaptation [23]) are the seemingly low levels of home adaptations in this group of people. Adaptations of the home were in place in less than a quarter of HD patients included in this study. Although this may be due to the fact that the minority of those surveyed in this study (at visit 1) were in the late stages of the disease and also that our data may not necessarily be representative of the HD population, further investigation is warranted. 

This analysis is not without limitations. It is important to acknowledge that the descriptive data presented here is specific to this fairly small sample (to date REGISTRY has enrolled over 6000 participants so our data is only reflective of approximately 8 % of the HD population). Additionally, almost all of the participants enrolled in REGISTRY are able to reach a HD clinic for annual assessments and on review it was clear that only a small proportion of our sample were in the late stages of the disease.  It is therefore likely that the data presented here is not representative of the most severely affected HD patients.

There will also be substantial differences across countries in terms of the support in the community that is available (i.e. service use is supply driven) and additionally there will clearly be inherent differences in health care systems between various European countries. The specific role that each member of the health care team may vary according to geographical region and this level of detail is not captured by the REGISTRY CSRI. Furthermore, there was no distinction between HD or non-HD related service use. It is difficult to say how much of the service use reported here is because of HD and there are many other factors which may lead a person to requiring formal health care services. 

Our data suggests a large reliance on informal care in the home in this group studied. At the first visit, half of all the HD patients in this study were in receipt of some manner of informal care in the home. It seems that informal carers will continue in this function up the very limits of their capacity and discussions with family members have highlighted some contributing factors. HD is seen as a family problem that ought to be managed by the involved family. Families trying to use available formal services encounter the problem of frequently changing care-givers and irregularity and restrictions in terms of the hours that the services can be provided. Such irregularity and frequent change is clearly not in agreement with the nature of HD and the person affected with HD. The HD patient is highly dependent on regularity and familiarity, making provision of community based care very challenging. Other studies of people with complex disability have suggested that where community care is limited, the burden of care often falls on the family and informal carers [24] [25]. Carer burden in this community should consequently be carefully considered by the healthcare team and where appropriate additional carer support should be provided. We suggest that use of routine rating scales employed in the clinical assessment may have some potential to identify high risk families where the informal care requirements are high.  From the data presented here, it is clear that the TFC had no predictive value on service receipt or on informal care hours whilst the UHDRS functional assessment scale (FAS) had reasonable predictive value on this variable. When individuals present with lower scores on the FAS, they appear to have higher informal care requirements and carer burden may additionally be higher. The FAS is an activity of daily living checklist with range from 0 to 25. The components of the checklist represent multiple domains of function including aspects of employment, finances, shopping, driving, housework, supervising children, personal activities of daily living, mobility and housework. This wide representation of domains may be one reason why the FAS seems to have better predictive value than TFC which is reflective of fewer domains. In this study, the FAS accounted for 30% of the variance in informal care hours by visit 3. In-depth investigation of suitable clinical scales and potential weighting of components may improve predictive value. Such targeted assessments could aid clinicians, funding agencies and policy makers in appropriate service planning and provision of co-ordinated care for HD patients. 

The data presented here is suggestive of the need for integrated and proactive care provision that addresses the requirements of the HD patients and their carers. It is clear that a method or a system to aid identification of factors indicative of the need for referral to formal services is required. Studies of the emotional experiences of family carers in HD and supportive care needs for people living with the disease highlight the high levels of emotional stress that can be associated with HD [4] [13]. It is important that care is individualised and specific to the needs of the HD patient. Availability of a consistent team of support workers and appropriate community support and carer support may facilitate management of the varying needs over time and prioritise referrals to services as needed [26] [4]. The high level of informal care reported here indicates that such a model of care is not yet systematically provided in the HD community and is an area that is in need of attention.   

## **Acknowledgements**

We would like to thank Dr Jonathan Gillard (Lecturer in Operational Research and Statistics, School of Mathematics, Cardiff University), Dr Lori Quinn (Honorary Research Fellow, Cardiff University), Dr Jenny Townhill (EHDN Language Co-ordination manager) and Professor David Cohen (Director and Professor of Health Economics. Health Economics and Policy Research Unit, University of Glamorgan) for their helpful comments related to the manuscript presented here. Many thank to the contributors at the study sites that have diligently collected and entered data into the REGISTRY database (see Appendix 1).   

**Address for correspondence**

Monica Busse

Email: busseme@cardiff.ac.uk

Department of Physiotherapy, School of Healthcare Studies, Cardiff University, Cardiff CF14 4XN, United Kingdom  

**Competing Interests**

The authors have declared that no competing interests exist. 

**Funding information **There were no major sources of funding for this work. Each author is supported by their current individual institution.  

**Appendix 1**

Contributors (study sites and individual members at the study sites) from the European Huntington’s disease Network to the data collected for analysis in this study are listed in alphabetical order by country below.  

AUSTRIA

Graz (LKH Graz, Abteilung für Psychiatrie): Raphael M. Bonelli; Brigitte Herranhof;  Anna Holl (formerly Hödl); Hans-Peter Kapfhammer; Michael Koppitz; Markus Magnet; Daniela Otti; Annamaria Painold; Karin Reisinger; Monika Scheibl; Karen Hecht; Sabine Lilek; Nicole Müller; Helmut Schöggl; Jasmin Ullah

Innsbruck (Universitätsklinik Innsbruck, Neurologie): Florian Brugger; Caroline Hepperger; Anna Hotter; Philipp Mahlknecht; Michael Nocker; Klaus Seppi; Gregor Wenning; Lisa Buratti; Eva-Maria Hametner; Christiane Holas; Anna Hussl; Katharina Mair; Werner Poewe; Elisabeth Wolf;  Anja Zangerl; Eva-Maria Braunwarth     

GERMANY

Aachen (Universitätsklinikum Aachen, Neurologische Klinik): Christoph Michael Kosinski; Daniela Probst; Christian Sass; Johannes  Schiefer; Christiane Schlangen; Cornelius J. Werner

Dinslaken (Reha Zentrum in Dinslaken im Gesundheitszentrums Lang): Herwig Lange

Dresden (Universitätsklinikum Carl Gustav Carus an der Technischen Universität Dresden, Klinik und Poliklinik für Neurologie):  Andrea Friedrich; Ulrike Hunger; Matthias Löhle; Simone Schmidt;  Alexander Storch; Annett Wolz; Martin Wolz

Freiburg (Universitätsklinik Freiburg, Neurologie): Johann Lambeck, Birgit Zucker

Hamburg (Universitätsklinikum Hamburg-Eppendorf, Klinik und Poliklinik für Neurologie): Kai Boelmans; Christos Ganos; Ute Hidding; Alexander Münchau; Michael Orth; Jenny Schmalfeld; Lars Stubbe; Simone Zittel

Heiligenhafen (Psychatrium Heiligenhafen): Walburgis Heinicke

Marburg KPP (Klinik für Psychiatrie und Psychotherapie Marburg-Süd): Bernhard Longinus

Marburg Uni (Universität Marburg, Neurologie): Jens Carsten Möller; Ida Rissling

München (Huntington-Ambulanz im Neuro-Kopfzentrum - Klinikum rechts der Isar der Neurologischen Klinik und Poliklinik der Technischen Universität München): Alexander Peinemann; Michael Städtler; Adolf Weindl

Münster (Universitätsklinikum Münster, Klinik und Poliklinik für Neurologie): Natalie Bechtel; Heike Beckmann; Stefan Bohlen; Eva Hölzner; Herwig Lange; Ralf Reilmann; Stefanie Rohm, Silke Rumpf ; Sigrun Schepers 

Taufkirchen (Isar-Amper-Klinikum - Klinik Taufkirchen (Vils)): Antonie Beister; Matthias Dose; Kathrin Hammer; Janina Kieni; Gabriele Leythaeuser; Ralf Marquard; Tina Raab; Sven Richter; Amina Selimbegovic-Turkovic; Caroline Schrenk; Michele Schuierer; Alexandra Wiedemann 

Ulm (Universitätsklinikum Ulm, Neurologie): Andrea Buck; Julia Connemann; Daniel Ecker; Carolin Eschenbach; Christine Held; Bernhard Landwehrmeyer; Franziska Lezius; Solveig Nepper; Anke Niess; Michael Orth, Sigurd Süßmuth, Sonja Trautmann; Patrick Weydt  

ITALY

Bologna (Università di Bologna, Dipartimento di Scienze Neuroloche - Clinica Neurologica): Sabrina Capellari; Pietro Cortelli; Roberto Gallasi; Roberto Poda; Giovanni Rizzo; Cesa Scaglione

Florence (Neurologia I- Unita' di Neurogenetica Departimento di Neurologia e Psichiatria, Universita' di Firenze): Elisabetta Bertini; Elena Ghelli, Claudia Mechi; Marco Paganini; Silvia Piacentini; Silvia Pradella; Anna Maria Romoli; Sandro Sorbi

Milan (Fondazione IRCCS Istituto Neurologico C. Besta, Milan):    Alberto Albanese; Stefano Di Donato; Cinzia Gellera; Silvia Genitrini; Caterina Mariotti; Daniela Monza; Lorenzo Nanetti; Domina Paridi; Paola Soliveri; Chiara Tomasello

Naples (Azienda Ospedaliera Universitaria Federico II - Dipartimento di Scienze Neurologiche): Giuseppe De Michele; Di Maio Luigi; Carlo Rinaldi; Cinzia Valeria Russo, Elena Salvatore; Tecla Tucci

Pozzilli (Neurogenetics Unit – IRCCS Neuromed): Milena Canella; Valentina Codella, Francesca De Gregorio, Nunzia De Nicola, Tiziana Martino; Mariella Simonelli; Ferdinando Squitieri 

Rome (Istituto di Neurobiologia e Medicina Molecolare CNR/ Istituto di Neurologia, Dipartimento di Neuroscienze/ CNR Istituto di Scienze e Tecnologie della Cognizione): Anna Rita Bentivoglio; Alfonso Fasano; Marina Frontali; Arianna Guidubaldi; Tamara Ialongo; Gioia Jacopini; Giovanna Loria; Carla Piano; Silvia Romano; Francesco Soleti; Maria Spadaro; Paola Zinzi  

NORWAY

Oslo University Hospital (Rikshospitalet, Dept. of Medical Genetics and Dep. of Neurology): Arvid Heiberg; Marleen R van Walsem, Jan Frich; Raghild Wehus; Olaf Aaserud; Nancy Borgerød

Oslo University Hospital (Ulleval, Dept of Medical Genetics and Department): Kathrine Bjørgo; Madelein Fannemel; Per Gørvell; Eirin Lorentzen; Susana Pro Koivisto; Lars Retterstøl; Torborg Overland; Bodil Stokke

Trondheim (St. Olavs Hospital): Inga Bjørnevoll; Sigrid Botne Sando  

POLAND

Gdansk (Specialistic Hospital, Gdansk Zaspa): Emilia Sitek; Jaroslaw Slawek; Witold Soltan.

Katowice (Silesian Medical University Katowice): Magdalena Boczarska-Jedynak; Barbara Jasinska-Myga; Gregorz Opala.

Krakow (Krakowska Akademia Neurologii): Krzysztof Banaszkiewicz; Andrzej Szczudlik; Monika Rudzińska; Magdalena Wójcik; Malgorzata Krawczyk.

Poznan (Medical University of Poznań): Anna Bryl; Anna Ciesielska; Aneta Klimberg; Jerzy Marcinkowski; Justyna Sempołowicz; Daniel Zielonka

Warsaw-MU (Medical University of Warsaw, Neurology): Piotr Janik; Anna Kalbarczyk; Hubert Kwiecinski; Zygmunt Jamrozik.

Warsaw-IPiN (Institute of Psychiatry and Neurology Dep. of Genetics, Dep. of Neurology): Jakub Antczak; Katarzyna Jachinska; Maria Rakowicz; Przemyslaw Richter; Danuta Ryglewicz; Grzegorz Witkowski; Elzbieta Zdzienicka; Jacek Zaremba.  

PORTUGAL

Lisbon-Santa Maria (Neurological Clinical Research Unit, Instituto de Medicina Molecular): Miguel Coelho; Leonor Guedes; Joaquim J Ferreira; Tiago Mendes; Tiago Mestre; Anabela Valadas.

Lisbon-Fernando Fonseca (Hospital Fernando Fonseca E.P.E., Serviço de Neurologia): Cristina Costa;  Ângela Timóteo.

Porto-Santo António (Hospital Geral de Santo António): Sara Cavaco; Joana Damásio; Marina Magalhães 

Porto-São João (Hospital São João, E.P.E.): Miguel Gago; Carolina Garrett; Maria Rosália Guerra.   

SPAIN

Badajoz (Hospital Infanta Cristina): Carmen Durán Herrera; Patrocinio Moreno Garcia

Granada (Hospital Universitario San Cecilio, Neurología): Francisco Barrero; Blas Morales 

Burgos (Servicio de Neurología Hospital General Yagüe): Esther Cubo; Natividad Mariscal; Jesús Sánchez

Madrid-Clinico (Hospital Clínico Universitario San Carlos): Rocío García-Ramos García; Purificacion Pin Quiroga; Clara Villanueva

Madrid RYC (Hospital Ramón y Cajal, Neurología): Mónica Bascuñana; Marta Fatás Ventura; Guillermo García Ribas; Justo García de Yébenes; José Luis López – Sendón Moreno, Patricia Trigo Cubillo 

Madrid FJD (Madrid-Fundación Jiménez Díaz): Pedro J García Ruíz, Asunción Martínez-Descals, María José Saiz Artiga; Vicenta Sánchez

Barcelona-Hospital Mútua de Terrassa : Miquel Aguilar Barbera; Dolors Badenes Guia; Laura Casas Hernanz ; Judit López Catena; Ana Rojo Sebastián, Pilar Quiléz Ferrer; Gemma Tome Carruesco

Barcelona-Bellvitge (Hospital Universitari de Bellvitge): Jordi Bas; Núria Busquets; Matilde Calopa

Palma (Hospital Son Dureta): Aranzazú Gorospe; Inés Legarda; Penelope Navas Arques; María José Torres Rodríguez, Barbara Vives

Sevilla (Hospital Virgen Macarena): Jose Manuel Garcia Moreno; José Chacón Peña; Luminita Dinca Avarvarei; Antonio Martín Bastida; Maria Fernández Recio; Luis Redondo Vergé; Violeta Sánchez Sánchez  

SWITZERLAND

Bern: Jean-Marc Burgunder; Yvonne Burgunder (Neurologische Klinik des Inselspitals); ; Alain Kaelin; Irene Romero; Michael Schüpbach; Sabine Weber Zaugg (Zentrum für Bewegungsstörungen, Neurologische Klinik und Poliklinik)  

UNITED KINGDOM

Aberdeen (NHS Grampian, Clinical Genetics Centre): Roisin Jack; Kirsty Matheson; Zosia Miedzybrodzka; Daniela Rae; Sheila Simpson; Fiona Summers; Alexandra Ure

Cardiff (The Institute of Medical Genetics, University Hospital of Wales): Monica Busse, Stephen Dunnett, Catherine Clenaghan, Ruth Fullham, Sarah Hunt, Lesley Jones, Una Jones, Hanan Khalil, Sara Minster, Michael Owen, Kathleen Price, Jenny Townshill, Anne Rosser.  

Edinburgh (Molecular Medicine Centre, Western General Hospital, Department of Clinical Genetics): Paul A. De Sousa; Maureen Edwards; Teresa Hughes (Scottish Huntington´s Association); Marie McGill; Pauline Pearson; Mary Porteous; Paul Smith (Scottish Huntington´s Association); Adam Zeman

Leicester (Leicestershire Partnership Trust, Mill Lodge): Colin Bourne; Carole Clayton; Heather Dipple; Jackie Clapton, Janet Grant; Diana Gross; Caroline Hallam; Julia Middleton; Ann Murch, Dawn Patino

London (St. Georges-Hospital): Michael Patton, Maria Patterson

Manchester (Genetic Medicine, University of Manchester, Manchester Academic Health Sciences Centre and Central Manchester University Hospitals NHS Foundation Trust): Natalie Arran; Jenny Callaghan; David Craufurd; Ruth Fullam; Liz Howard; Susan Huson; Emma Oughton; Lucy Partington-Jones;); Julie Snowden; Andrea Sollom; Cheryl Stopford; Jennifer Thompson; Iris Trender-Gerhad; Nichola Verstraelen (formerly Ritchie); Leann Westmoreland

Sheffield (The Royal Hallamshire Hospital): Oliver Bandmann; Alyson Bradbury, Paul Gill, Helen Fairtlough, Kay Fillingham, Isabella Foustanos; Kirsty O’Donovan; Nadia Peppa, Katherine Tidswell, Oliver Quarrell             

## References

[ref-482334756] Walker FO. Huntington's disease. Lancet. 2007 Jan 20;369(9557):218-28. Review. 17240289 10.1016/S0140-6736(07)60111-1

[ref-652509782] McCabe MP, Roberts C, Firth L. Satisfaction with services among people with progressive neurological illnesses and their carers in Australia. Nurs Health Sci. 2008 Sep;10(3):209-15. 18786063 10.1111/j.1442-2018.2008.00399.x

[ref-990648574] Nance MA. Comprehensive care in Huntington's disease: a physician's perspective. Brain Res Bull. 2007 Apr 30;72(2-3):175-8. Epub 2006 Nov 20. Review. 17352943 10.1016/j.brainresbull.2006.10.027

[ref-503210668] Dawson S, Kristjanson LJ, Toye CM, Flett P. Living with Huntington's disease: need for supportive care. Nurs Health Sci. 2004 Jun;6(2):123-30. 15130098 10.1111/j.1442-2018.2004.00183.x

[ref-1397034122] Wheelock VL, Tempkin T, Marder K, Nance M, Myers RH, Zhao H, Kayson E, Orme C, Shoulson I; Huntington Study Group. Predictors of nursing home placement in Huntington disease. Neurology. 2003 Mar 25;60(6):998-1001. 12654967 10.1212/01.wnl.0000052992.58107.67

[ref-1772047877] Aubeeluck A, Wilson E. Huntington's disease. Part 1: essential background and management. Br J Nurs. 2008 Feb 14-27;17(3):146-51. Review. 18414253 10.12968/bjon.2008.17.3.28402

[ref-2018362817] Moskowitz CB, Marder K. Palliative care for people with late-stage Huntington's disease. Neurol Clin. 2001 Nov;19(4):849-65. Review. 11854103 10.1016/s0733-8619(05)70050-5

[ref-3920981270] Simpson SA. Late stage care in Huntington's disease. Brain Res Bull. 2007 Apr 30;72(2-3):179-81. Epub 2006 Nov 16. Review. 17352944 10.1016/j.brainresbull.2006.10.022

[ref-1861122207] Skirton H, Glendinning N. Using research to develop care for patients with Huntington's disease. Br J Nurs. 1997 Jan 23-Feb 12;6(2):83-90. 9116444 10.12968/bjon.1997.6.2.83

[ref-2345126500] Veenhuizen RB, Tibben A. Coordinated multidisciplinary care for Huntington's disease. An outpatient department. Brain Res Bull. 2009 Oct 28;80(4-5):192-5. Epub 2009 Jul 1. 19576265 10.1016/j.brainresbull.2009.06.017

[ref-2006398220] Aubeeluck A, Moskowitz CB. Huntington's disease. Part 3: family aspects of HD. Br J Nurs. 2008 Mar 13-26;17(5):328-31. Review. 18414297 10.12968/bjon.2008.17.5.28830

[ref-2226054571] Aubeeluck A. Caring for the carers: quality of life in Huntington's disease. Br J Nurs. 2005 Apr 28-May 11;14(8):452-4. Review. 15924027 10.12968/bjon.2005.14.8.17929

[ref-172999484] Williams JK, Skirton H, Paulsen JS, Tripp-Reimer T, Jarmon L, McGonigal Kenney M, Birrer E, Hennig BL, Honeyford J. The emotional experiences of family carers in Huntington disease. J Adv Nurs. 2009 Apr;65(4):789-98. Epub 2009 Feb 9. 19228233 10.1111/j.1365-2648.2008.04946.x PMC3807601

[ref-4194119704] Department of Health. The National Service Framework for long term conditions. 2005. London, UK.

[ref-1491090897] Chisholm D, Knapp MR, Knudsen HC, Amaddeo F, Gaite L, van Wijngaarden B. Client Socio-Demographic and Service Receipt Inventory--European Version: development of an instrument for international research. EPSILON Study 5. European Psychiatric Services: Inputs Linked to Outcome Domains and Needs. Br J Psychiatry Suppl. 2000;(39):s28-33. 10945075 10.1192/bjp.177.39.s28

[ref-138785249] Beecham, J and Knapp, M. Costing Psychiatric Interventions, in Measuring Mental Health Needs, Eds.Thornicroft G, Brewin C & Wing JK. 1992. Gaskell: London.

[ref-768890800] Schneider J, Hallam A, Murray J, Foley B, Atkin L, Banerjee S, Islam MK, Mann A. Formal and informal care for people with dementia: factors associated with service receipt. Aging Ment Health. 2002 Aug;6(3):255-65. 12217094 10.1080/13607860220142486

[ref-3247399554] Unified Huntington's Disease Rating Scale: reliability and consistency. Huntington Study Group. Mov Disord. 1996 Mar;11(2):136-42. 8684382 10.1002/mds.870110204

[ref-1823827456] Shoulson I, Fahn S. Huntington disease: clinical care and evaluation. Neurology. 1979 Jan;29(1):1-3. 154626 10.1212/wnl.29.1.1

[ref-1193800918] Orth M; European Huntington's Disease Network, Handley OJ, Schwenke C, Dunnett SB, Craufurd D, Ho A, Wild EJ, Tabrizi SJ. Observing Huntington's Disease: the European Huntington's Disease Network's REGISTRY. PLoS Curr. 2010 Sep 28;2. pii: RRN1184. 20890398 10.1371/currents.RRN1184 PMC2947793

[ref-519413508] Currie G, Kerfoot KD, Donaldson C, Macarthur C. Are cost of injury studies useful? Inj Prev. 2000 Sep;6(3):175-6. 11003180 10.1136/ip.6.3.175 PMC1730644

[ref-3690011302] Busse ME, Khalil H, Quinn L, Rosser AE. Physical therapy intervention for people with Huntington disease. Phys Ther. 2008 Jul;88(7):820-31. Epub 2008 May 8. 18467429 10.2522/ptj.20070346

[ref-1271473156] Freeman JA, Thompson AJ. Community services in multiple sclerosis: still a matter of chance. J Neurol Neurosurg Psychiatry. 2000 Dec;69(6):728-32. 11080223 10.1136/jnnp.69.6.728 PMC1737174

[ref-1416594504] Phillips VL. Community care for severely disabled people on low incomes. BMJ. 1995 Oct 28;311(7013):1121-3. 7580705 10.1136/bmj.311.7013.1121 PMC2551053

[ref-1842333834] Williams MH, Bowie C. Evidence of unmet need in the care of severely physically disabled adults. BMJ. 1993 Jan 9;306(6870):95-8. Retraction in: BMJ. 1998 Jun 6;316(7146):1700. 8435649 10.1136/bmj.306.6870.95 PMC1676639

[ref-3929845240] Bernard S, Aspinal F, Gridley K, Parker G. Integrated policy making in England for adults with long-term neurological conditions (LTNCs): some preliminary findings from a scoping study. Int J Integr Care. 2008 Jul 14;8:e60. 18695746 10.5334/ijic.247 PMC2504699

